# A case study of repetitive transcranial magnetic stimulation for cryptococcal meningitis combined with cognitive impairment

**DOI:** 10.3389/fnhum.2022.1061916

**Published:** 2022-12-16

**Authors:** Yuanbiao Liu, Lei Fan, Xinlin Jiang, Yi Lu, Yige Li

**Affiliations:** ^1^Department of Rehabilitation Medicine, Second Affiliated Hospital of Nanjing Medical University, Nanjing, China; ^2^Physical Medicine and Rehabilitation Unit, Nanjing Medical University, Nanjing, China

**Keywords:** cryptococcal meningitis, repetitive transcranial magnetic stimulation, cognitive impairment, rehabilitation, inflammatory cytokines

## Abstract

Cryptococcal meningitis (CM) is a central nervous system disease caused by a novel *Cryptococcus* infection that leads to subacute or chronic inflammatory changes in the nervous system. In this study, we present the case of a woman aged 72 years with CM and severe cognitive impairment and disabilities. The cognitive assessment indicated that, although her cognitive function was impaired, especially executive function, it largely improved after receiving anti-infectious and repetitive transcranial magnetic stimulation, which can alter the membrane potential of the cortical nerve cells by triggering long-term potentiation and depression, modulating and releasing hormones, reducing the level of neuroinflammatory and peripheral blood cytokines, promoting nerve regeneration and synaptic remodeling, and changing the activity of the neural circuitry of the dorsolateral prefrontal cortex. We argue that this case provides a novel method of treatment for patients with CM in conjunction with cognitive impairments.

## Introduction

Cryptococcal meningitis (CM) is a central nervous system disease caused by a novel mesophilic *Cryptococcus* that can penetrate the blood–brain barrier through a specific mechanism (Wang et al., 2013). As the primary target of the novel *Cryptococcus* is the central nervous system, *Cryptococcus* infections can easily result in subacute or chronic inflammatory changes in the central nervous system. Furthermore, long-term use of immunosuppressants, immunodeficiencies, severe trauma, and systemic chronic diseases is associated with a high infection rate, and people with a normal immune function who carry individual susceptibility factors are also prone to infection. Studies showed that the complement system and glucuronide xylose rush glycan are related to individual susceptibility (Vecchiarelli et al., 2013). In addition to the common symptoms of CM, which are caused by increased intracranial pressure (ICP), infection with the novel *Cryptococcus* can cause brain parenchymal damage, with corresponding clinical manifestations including hemiplegia, epilepsy, mental disorders, and cognitive impairment (Mao et al., 2017). The diagnostic accuracy can be improved by combining repeated cerebrospinal fluid (CSF) bacterial smears, latex agglutination tests, and imaging.

Anti-infective medication involves the administration of flucytosine, and amphotericin B with high sensitivity is prioritized in the treatment of CM. Supportive therapy according to symptoms, such as dehydration, lowering ICP, and neuroprotection, can yield better outcomes. Additionally, it is crucial to initiate early and proper rehabilitation for dysfunction after infection. A study on 27 patients with CM found significantly worse executive function performance in patients with ventriculomegaly (VM) than in those without VM (Traino et al., 2019). Another study showed that the related inflammatory cytokines [growth-related oncogenes; interleukin (IL)-10, IL-2, and IL-8; macrophage inflammatory protein-1β; and tumor necrosis factor (TNF)-α] were negatively correlated with the Montreal Cognitive Assessment (MoCA) score of patients, whereas the MoCA score was significantly positively correlated with dementia-related factors (αβ42 and total tau) (Tao et al., 2022). In addition, Lu et al. reported an indirect relationship between neuropsychological performance and CSF antigen titer: the higher CSF cryptococcal-antigen titer on admission may be associated with poorer cognitive function (Lu et al., 2011).

In addition to conventional drug therapy and cognitive therapy, repetitive transcranial magnetic stimulation (rTMS) has been widely used in clinical practice in recent years to improve the cognitive function of patients with cognitive impairment. rTMS can reduce the levels of inflammatory cytokines such as IL-8, IL-10, and TNF-α (Xu, 2021). In this study, a patient with CM combined with cognitive impairment as the main manifestation underwent rTMS-based therapy, and satisfactory clinical effects were obtained.

## Clinical summary

**Figure 1:**
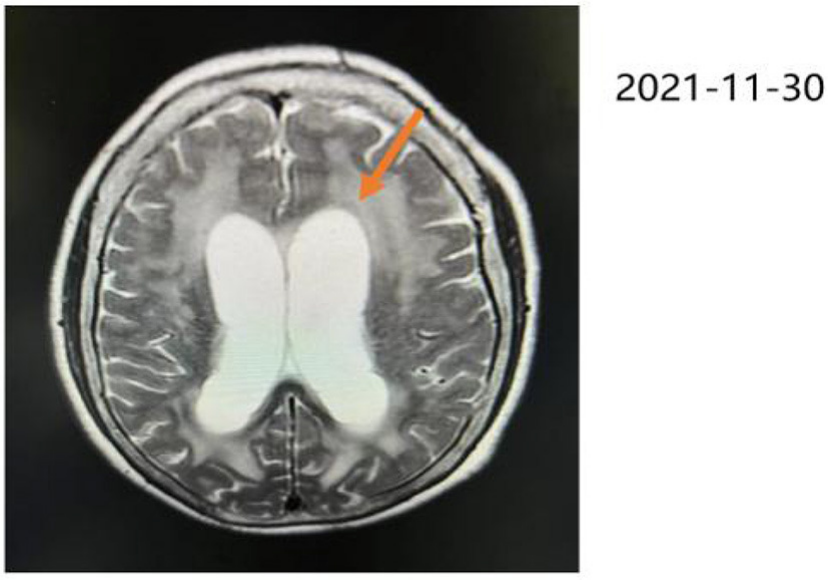
Brain MRI in T2-weighted sequence, taken on 30 November 2021, revealing hydrocephalus, ischemic changes in the bilateral frontoparietal occipital lobe, age-related brain changes, and ventriculomegaly where indicated by the orange arrow.

**Figure 2:**
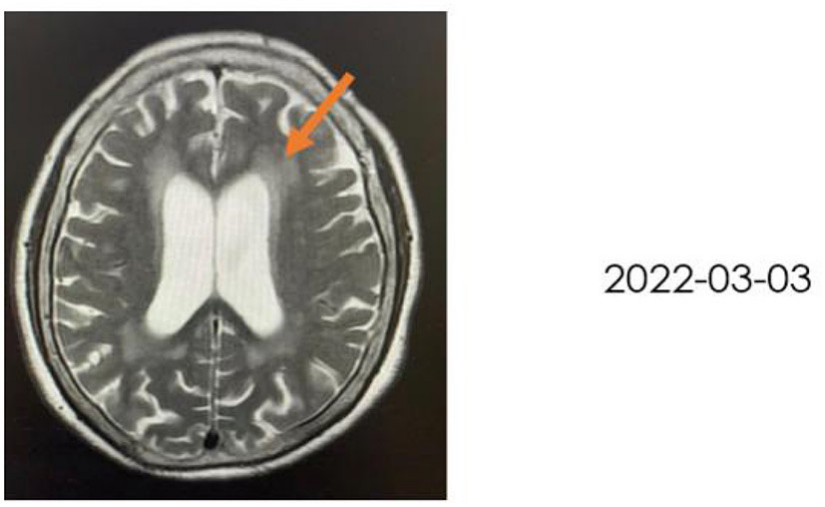
Brain MRI took on 3 March 2022, revealing significantly reduced ventricles.

A 72-year-old woman with a middle school education who presented with headache, dizziness, nausea, and vomiting was admitted to a local hospital. In August 2021, she was diagnosed with tuberculous meningitis and underwent antituberculosis therapy. However, her symptoms did not disappear. After being discharged from the local hospital, she visited the neurology department of our hospital. CSF analysis and India ink staining of the CSF revealed *Cryptococcus*, and she was diagnosed with CM. She received antifungal therapy [fluconazole (10 mg/kg/day) and 5-fluorocytosine (100 mg/kg/day)] for almost 2 months before being affected by rapidly progressive dementia caused by central nervous system infection. On 25 November 2021, she began to show signs of confusion, an inability to speak, limited limb movements, an inability to walk, an inability to eat independently, signs of nausea without vomiting, and signs of incontinence, but she could still open her eyes when called and lacked limb convulsions. She was then hospitalized in our department. A lumbar puncture and CSF analysis were performed, which revealed an ICP of >200 mmH_2_O, a white blood cell (WBC) count of 7.4 cells/μl. a glucose level of 1.83 mmol/L, a protein level of 2,496 mg/L, and a chlorine level of 112.3 mmol/L. which suggested an inflammation. India ink staining of the CSF revealed *Cryptococcus* and CSF culture revealed *Cryptococcus neoformans*. Moreover, multiple intracranial lesions with meningeal enhancement were observed on brain magnetic resonance imaging (MRI; Figure 1). During hospitalization, the consciousness and mental state of the patient gradually improved after antifungal treatment with fluconazole and fluoropyrimidine, and cognitive function assessments revealed that the cognitive dysfunction of the patient persisted (Mini-Mental State Examination: 11 points, MoCA: 7 points); hence, the patient was referred to the Department of Rehabilitation Medicine for appropriate treatment while she demonstrated a weakness of the lower limbs and gait disturbance. After 2 weeks of receiving rTMS treatment, the condition of the patient was reevaluated, which revealed that all cognitive functions had significantly improved. The patient was conscious and responded slowly. Her ICP reached >200 mmH_2_O, and she had a WBC count of 11.83 cells/μl, a glucose level of 2.37 mmol/L, a protein level of 2,111 mg/L, and avchlorine level of 114.6 mmol/L. Additionally, the brain MRI images taken on 03 March 2022 revealed a significant reduction in the ventricles (MRI; Figure 2). Though the CSF culture was negative and cognitive function improved after rTMS therapy, we did not found a significant decline in inflammatory signals.

Written informed consent was obtained from the individual for the publication of any potentially identifiable images or data included in the article.

## Assessment method

The Beijing version of the MoCA was used to screen the cognitive function of patients, which includes 11 examination items in eight cognitive domains (attention and concentration, executive function, memory, language, visual structural skills, abstract thinking, calculation, and orientation).

The Loewenstein Occupational Therapy Cognitive Assessment (LOTCA) was used to evaluate the functions of patients such as orientation, perception, optic-motor organization, and thinking operation.

The Rivermead Behavioral Memory Test (RBMT) was used to measure the behavioral memory function in daily life, which includes 12 items (including remembering names, remembering concealed objects, remembering appointments, picture recognition, story immediate recall, remembering directions and dates, routes delayed recall, and letters delayed recall).

## Treatments

First, the medications of the patients were continued. According to previous studies, the effect of medications is limited. Other studies indicated that cognitive impairment is the primary complication in individuals with CM. When only anti-infective medications are taken, it takes >1 month to observe signs of improvement in cognitive dysfunction (Mao et al., 2017). To further improve the treatment effects, we added rTMS therapy to the previous treatment protocol. The patient was informed of the risks and advantages of rTMS therapy prior to treatment, and consent was obtained from the patient and her family before initiating the treatments. For rTMS therapy, we used the butterfly coil D-B80 with a stronger focus (Mag Pro R30; Mag Venture; Denmark). The motor threshold (MT) of the patient was measured during the initial treatment. The stimulation site was selected as the left dorsolateral prefrontal cortex (DLPFC) of the patient. The stimulation intensity was set to 100% MT, and the stimulation frequency was 20 Hz. Each training session involved 20 stimulation pulses with a total of 50 trains, and the interval between each train was 30 s. A total of 1,000 stimulation pulses were used in a single treatment, which took approximately 25 min per session. The patient was treated for 14 consecutive days, with one treatment session per day.

## Summary

Before rTMS treatment, the cognitive scale assessment results of the patient (Tables 1–3) indicated that the patient had severe cognitive impairment, and the MoCA assessment results revealed that the cognitive function of the patient was severely impaired; visuospatial and executive functions, object naming function, and abstract thinking function were severely reduced; and language function, short-term memory, and delayed recall function were impaired. The findings of the tests conducted using LOTCA and RBMT were identical in that the patients had a functional impairment and dysfunction in thinking operations, learning imitation, attention, and concentration. The scores on the aforementioned scales met the requirements for significant cognitive impairment.

**Table 1:** The assessment results of MoCA.

**Project**	**Visuospatial and executive function**	**Object naming function**	**Attention**	**Language**	**Thinking function**	**Delayed memory**	**Orientation**	**Total**
Before	0/5	0/3	1/6	1/3	0/2	1/5	4/6	7/30
After	4/5	2/3	5/6	2/3	1/2	3/5	5/6	22/30

**Table 2:** The assessment results of LOTCA.

**Project**	**Orientation**	**Visual perception**	**Spatial perception**	**Motor praxis**	**Visuomotor organization**	**Logic**	**Attention and concentration**	**Total**
Before	2/16	8/16	6/12	7/12	13/28	9/35	2/4	47/123
After	12/16	11/16	11/12	11/12	21/28	23/35	3/4	92/123

**Table 3:** The assessment results of RBMT (delayed recall/immediate recall).

**Project**	**First and second names (D)**	**Belongings (D)**	**Appointments (D)**	**Route** **(I)**	**Route** **(D)**	**Novel task (I)**	**Novel task (D)**	**Orientation**	**Time**	**Face recognition**	**Picture recognition**	**Total**
Before	0/2	1/1	0/1	0/1	0/1	1/1	0/1	0/1	0/1	0/1	0/1	2/12
After	2/2	1/1	1/1	0/1	0/1	1/1	1/1	1/1	0/1	1/1	1/1	9/12

The patient underwent a second evaluation of her cognitive performance after 2 weeks of rTMS treatment. The results showed that the cognitive function of the patient was greatly improved, and each subscore of the evaluation was very close to normal. The comprehensive analysis of assessment results and clinical observations revealed that the visuospatial function of the patient was significantly improved, and she could distinguish between her left and right limbs and identify the corresponding relationship between the front and the back and the left and the right of a picture. Meanwhile, the executive function, which includes drawing and imitating sketching, also improved. Time cognition and language function also significantly improved, and the patient could accurately express the current year, month, and season; tell the current time according to the clock; and name simple things. Furthermore, the memory function of the patient significantly improved; she was able to remember some basic phrases and complex routes and could execute simple recall tasks after a delay. However, the recall of complex tasks still required further improvement. In terms of learning and attention, the patient could learn how to use a calculator and imitate and copy graphics; in terms of logical thinking, the patient could classify simple objects and respond to straightforward logical questions.

## Discussion

The clinical symptoms of CM are diverse, and they are often accompanied by irregular fever, increased ICP, blurred vision, gait disturbance, headache, and vomiting. Inflammation can also cause cranial nerve and brain parenchymal damage, leading to a series of problems such as cognitive dysfunction. Due to the insidious onset of cognitive dysfunction and the less prominent manifestation of single cognitive dysfunction, CM is easily misdiagnosed or ignored clinically. As a result, prompt treatment can be delayed, which negatively impacts the prognosis of the patient.

Once the cognitive function is disrupted, abilities such as attention, speech, and execution are limited and impaired to varying degrees, which has a significant impact on the daily lives of the patients. Currently, medication therapy or corresponding cognitive training is typically used to treat cognitive impairment. However, it usually takes a prolonged time to achieve therapeutic effects, and the adverse effects of the medications are significant. Hence, it is crucial to identify innovative, effective, and side-effect-free therapy options. In recent years, TMS has shown significant clinical effects in improving post-stroke cognitive impairment (PSCI) (Hu et al., 2012). It generates induced currents through stimulation coils in specific brain regions and stimulates the cortex. Thus, it can achieve transmembrane, transcortical, and cortical networks, altering the membrane potential of cortical nerve cells by triggering synaptic long-term potentiation (LTP) and long-term depression (LTD). The distant effects of rTMS therapy may result in functional modifications of the distant cortex at the stimulation site so that the goals of in-depth treatment could be accomplished (Liu et al., 2017). Previous studies suggested that altering the membrane potential of the cortical nerve cells by triggering LTP and LTD, modulating and releasing hormones, reducing the level of neuroinflammatory and peripheral blood cytokines, promoting nerve regeneration and synaptic remodeling, and changing the activity of the neural circuitry of the DLPFC may be involved in the mechanism of rTMS.

Several previous studies showed that rTMS can modulate the release of neurohormones, such as regulation of dopamine synthesis and release, and restore the function of cerebral cortical networks. A study using animal models reported that rTMS could upregulate the expression of GAP-43, Syp, and neurotrophic factors, suggesting that it can promote nerve regeneration and synaptic remodeling (Koch et al., 2014). In addition, among healthy young people, multiple sessions of high-frequency rTMS over the left DLPFC can increase resource recruitment of cognitive control and enhance resource efficiency, thus deploying for conflict resolution during multiple stages of cognitive control processing. The left dorsal frontal lobe area is involved in cognitive and emotional function (Li, 2017). Various studies reported that the application of rTMS to the left DLPFC improves emotional and cognitive functions. Furthermore, studies on the neuropathological analysis of AD brains revealed that neuroinflammation is an important driving force for neurodegeneration and AD progression (Motta et al., 2020), which is associated with significantly increased levels of CSF IL-4, IL-6, IL-8, and granulocyte colony-stimulating factor. A previous study suggested that the levels of CRP, TNF-α, IL-1β, IL-6, and IL-8 in the peripheral blood of patients with mild cognitive impairment (MCI) were higher than those in the normal population. A previous study also showed that rTMS therapy could decrease the levels of inflammatory cytokines (such as TNF-α, IL-2, IL-1β) (Li and Cheng, 2018; Lu et al., 2022), which can cross the blood–brain barrier, and high levels can affect the normal metabolism of monoamine neurotransmitters in the brain. In this case, the use of rTMS therapy on the patient led to better performance on the cognitive scale assessment results of the patient, but we did not observe a significant correlation between cognitive function and a change in inflammatory cytokines. A limitation of the current study is that neuroinflammatory biomarkers of the patients were not examined, as peripheral biomarkers are easily affected by other factors such as infectious disease. As a limitation, additional correlations, such as biomarkers and inflammatory cytokines, will require further studies. In addition, rTMS can also be complemented with the assessment of a putative marker of central cholinergic transmission. Short latency afferent inhibition (SAI) is a paired-pulse TMS protocol involving the inhibition of motor-evoked potentials by afferent sensory impulses. Previous findings pointed out that SAI is a putative marker of central cholinergic transmission, which can be modulated by ongoing recognition memory, specifically for the retrieval process (Sun and Zhao, 2021). Taken together, it could be applied not only in healthy subjects but also in patients with neurodegenerative diseases, such as Parkinson's or Alzheimer's disease, whose SAI is significantly impaired (Bonnì et al., 2017).

Combined with clinical data, the possible mechanisms promoting the recovery of cognitive functions in different dimensions after CM through rTMS treatment were explored, as discussed below.

*Memory function* can be divided into instantaneous memory, short-term memory, and long-term memory according to the time of information extraction, and these three types of memories are related to each other. High-frequency rTMS has been demonstrated to increase delayed memory function in patients with MCI, reduce memory encoding time, and boost memory storage modules in healthy individuals (Martorana et al., 2009). According to functional MRI results, high-frequency rTMS can enhance memory performance by boosting the functional connectivity of the hippocampal-cortical network (Floel and Cohen, 2007). In parallel, animal studies showed that rTMS could improve synaptic ultrastructure in the hippocampal CA1 region and enhance the mRNA and protein expression of brain-derived neurotrophic factor, N-methyl-D-aspartic acid receptor, and synaptophysin so that it could further affect synaptic plasticity (Wang and Voss, 2015), enhance the modulation of hippocampus function *via* the dopaminergic system, and increase the memory storage (Floel and Cohen, 2007). The findings of our analysis showed that the delayed and instantaneous memory of the patient had improved to some degree, indicating that high-frequency rTMS may have a certain curative effect on the improvement of memory function.

*Time perception* and memory function are inseparable. Objective data are transformed into a time interval representation through the memory process, and decisions are made by comparing the current time interval with the time image stored in long-term memory. The dorsolateral prefrontal cortex, the cerebellum, the basal ganglia, and the auxiliary motor areas are the primary brain regions involved in the cognitive processing of time perception (Wang et al., 2015). Previous research demonstrated that the DLPFC, which serves as the primary memory of the internal clock, receives input from the cerebellum and the basal ganglia to create a time interval representation and encodes the temporal representation before storing it in memory (Yin et al., 2008). Additionally, some researchers discovered that time perception within 1 s is controlled by the cortico-basal ganglia-thalamic network. This was discovered by stimulating the cerebellum site (Gironell et al., 2005). Other studies in this area revealed that stimulating the primary audiovisual cortex of the parietal lobe could positively affect audiovisual time perception (Lee et al., 2007). Thus, when investigating the stimulation targets for time cognition, a comprehensive analysis of the time perception conditions of the patient should be done to determine the optimal stimulation site for treatment. If necessary, stimulation of multiple sites can be performed to enhance these effects.

*Visuospatial ability*. The three cognitive skills of attentiveness, orientation, and execution make up the visuospatial ability, which indicates the ability to point and focus on spatial information. Functional imaging of the human brain has revealed that functional regions such as the dorsolateral frontal cortex, the anterior cingulate gyrus, and the parietal cortex play a major role in the activation of visuospatial networks (Mifsud et al., 2014). In one study, subjects underwent theta-burst repetitive TMS (cTBS) to stimulate their bilateral dorsolateral prefrontal cortex and inflict “virtual damage.” Patients whose right DLPFC was stimulated displayed decreased alertness and executive function, whereas those whose left DLPFC was stimulated displayed decreased orientation function. Hence, it can be assumed that the bilateral DLPFC are both the centers for the initiation and regulation of the visuospatial attention process (Sturm and Willmes, 2001). The high-frequency rTMS employed in this study had an excitatory effect on the neural activity of the stimulation site as opposed to the inhibitory function of cTBS. The distant effect induced by TMS also resulted in the excitation of brain activity in the posterior parietal lobe, in addition to the LTP effect of the left DLPFC. The frontoparietal loop of the conductive pathway of the patient, which is primarily concerned with determining the spatial location of items, is further activated by the excitatory action (Xu et al., 2013). In this case, we selected high-frequency stimulation to activate the left DLPFC of the patient according to the theory of the interhemispheric competition model of brain injury. It could be speculated that the right DLPFC was in a suppressed state at this time. However, the second evaluation results of this case demonstrated that the actual effect is contradictory to the prognosis, in which inhibition of the right DLPFC did not cause a decline in vigilance and executive function. In turn, the concentration and executive function performance of the patient greatly improved after TMS. This outcome currently fails to explain the mechanism, and further investigation is required.

*Language* is a unique high-level cognitive function in human beings. According to functional MRI studies based on several baseline tasks, the overall functional network of the frontal-parietal-subcortical regions is more functionally employed than Wernicke's and Broca's areas for language processing, where the dorsolateral prefrontal cortex plays a major role in the process of detecting incorrect language information and suppressing nontarget languages (Yantis et al., 2002). In this case, during the second cognitive evaluation of the patient, the language function had significantly improved, and the usage of incorrect and random words had significantly decreased. It may be inferred that high-frequency rTMS could encourage the rearrangement of the language function of the patient and enhance the connectivity between synapses in the neural networks of language centers. According to a study, white matter integration around the left hemisphere is strengthened after high-frequency stimulation of the frontal lobe. Simultaneously, the functional connection between the white matter and the reward processing center of the brain, such as the hippocampus and the caudate nucleus, is enhanced (Hu, 2015).

*Executive function*. To ensure the general control of specific targets by the cognitive system in a more flexible and optimized manner, the executive function, as an important high-level cognitive processing of individuals, is in charge of coordinating various cognitive resources and procedures in the process of completing complex cognitive tasks. The dorsolateral prefrontal cortex is an important center of the executive control network. Some studies showed that healthy individuals could gain better results in executive function, as measured by the Stroop test, after high-frequency rTMS (Martorana et al., 2009). Other studies also demonstrated that the Stroop test scores were significantly improved after 4 weeks of high-frequency rTMS stimulation on the left dorsolateral prefrontal lobe in individuals with PSCI compared with those before treatment and 2 weeks after treatment (Vanderhasselt et al., 2006). In this case, the executive function of the patient significantly improved after consecutive rTMS therapy. In addition, elevated neurometabolic research suggests that biological markers that affect the cognitive executive function of a person, such as N-acetyl aspartate and choline complexes, are markedly elevated in patients with executive dysfunction (Wang and Voss, 2015). In conclusion, high-frequency rTMS can effectively regulate synaptic plasticity, enhance neural excitability, induce arousal of the cortex in cerebral function areas, and activate networks of executive control in the frontal lobe.

According to related studies on TMS paired with event-related potentials, the amplitudes of N2 and N450, two significant components closely associated with cognitive control and conflict monitoring, can be enlarged by rTMS in the course of completing tasks, while incorrect electroencephalogram signals can be modified by rTMS during task execution (Yin et al., 2018). The NoGo-N2 potential, which reflects the number of cognitive resources used in the early stages of response inhibition, is one of the waveforms implicated in response inhibition. According to a previous study, high-frequency rTMS can stimulate the entire neural circuit involved in response inhibition and significantly improve cognitive function in patients with deficits in response inhibition (Zhou et al., 2017).

## Conclusion

Anti-infective therapy should be primarily addressed for the treatment of CM so that the cognitive impairments and other symptoms of the patients might be improved after controlling the infection. Additionally, rTMS therapy might improve the cognitive performance of the patient in different categories, including memory function, time perception, visuospatial cognition, speech, and executive function. This case provides a novel treatment method for patients with CM in conjunction with cognitive impairment, and our findings serve as a foundation for future clinical investigations. However, it is essential to increase the patient sample size to conduct a thorough analysis because of the limitations of case studies on individual differences. Finally, further exploration and research could be conducted on the selection of precise stimulation targets and stimulation parameters in rTMS, as well as analysis combined with imaging examinations, neurophysiology, and other clinical techniques.

## Data availability statement

The raw data supporting the conclusions of this article will be made available by the authors, without undue reservation.

## Ethics statement

Written informed consent was obtained from the individual for the publication of any potentially identifiable images or data included in the article.

## Author contributions

YLi: data curation, formal analysis, visualization, writing—original draft, and writing—review and editing. LF: data curation, formal analysis, investigation, visualization, writing—original draft, and writing—review and editing. XJ: data curation, formal analysis, investigation, writing—original draft, and writing—review and editing. YLu: conceptualization, data curation, formal analysis, supervision, validation, visualization, and writing—review and editing. YL: data curation, investigation, writing—original draft, and writing—review and editing. All authors contributed to the article and approved the submitted version.
